# Osgood-Schlatter Lesion Removed Arthroscopically in an Adult Patient

**DOI:** 10.7759/cureus.7362

**Published:** 2020-03-22

**Authors:** George Tsakotos, Dimitrios A Flevas, Grigorios G Sasalos, Leonardos Benakis, Anastasios V Tokis

**Affiliations:** 1 Anatomy, School of Medicine, National and Kapodistrian University of Athens, Athens, GRC; 2 Arthroscopy and Orthopaedic Surgery, Metropolitan Hospital, Athens, GRC

**Keywords:** arthroscopy, knee, ununited ossicle, osgood-schlatter disease

## Abstract

Osgood-Schlatter disease is a traction apophysitis of the tibial insertion of the patellar tendon. It consists one of the most common causes of knee pain in adolescents and usually presents in young males and it is considered a self-limiting condition. Although the symptoms disappear after the closure of the growth plate in most cases, in some patients they may persist. A variety of conservative treatments are used in most cases, however surgical intervention can be successful for patients who have intolerable symptoms. Most surgical options of the Osgood-Schlatter disease include open procedures, while arthroscopic or direct bursoscopic excision has been reported. We believe that the arthroscopic removal of an unresolved Osgood-Schlatter might be the most appropriate treatment for this condition, and we present a case of a male patient with an ununited ossicle due to an Osgood-Schlatter lesion, which was removed arthroscopically using a multidirectional arthroscope and a motorized semi-hooded barrel burr.

## Introduction

Osgood-Schlatter disease was first described in 1903 and is a traction apophysitis of the tibial insertion of the patellar tendon caused by the repetitive strain on the quadriceps femoris muscle [1]. It consists one of the most common causes of knee pain in adolescents and usually presents in young males aged 10 to 14 years, with a bilateral knee ratio of 25% to 33%. It is a self-limiting condition, with resolution of symptoms in about 90% of cases with or without some form of conservative treatment and although the symptoms disappear after the closure of the growth plate in most cases, in some patients they may persist [2-5].

In most cases conservative treatments, which include rest, lidocaine injection, steroid injection, cylinder casts and infrapatellar straps, are adequate [6]. However, although conservative management has been conventionally favored, surgical intervention can be successful for patients who have intolerable symptoms [7]. Most surgical options of the Osgood-Schlatter disease include open procedures, while arthroscopic or direct bursoscopic excision has been reported [6,7-11,12,13].

Hereby we report a case of a male patient with an ununited ossicle due to an Osgood-Schlatter lesion, which was removed arthroscopically using a multidirectional arthroscope and a motorized semi-hooded barrel burr.

## Case presentation

**Figure 1:**
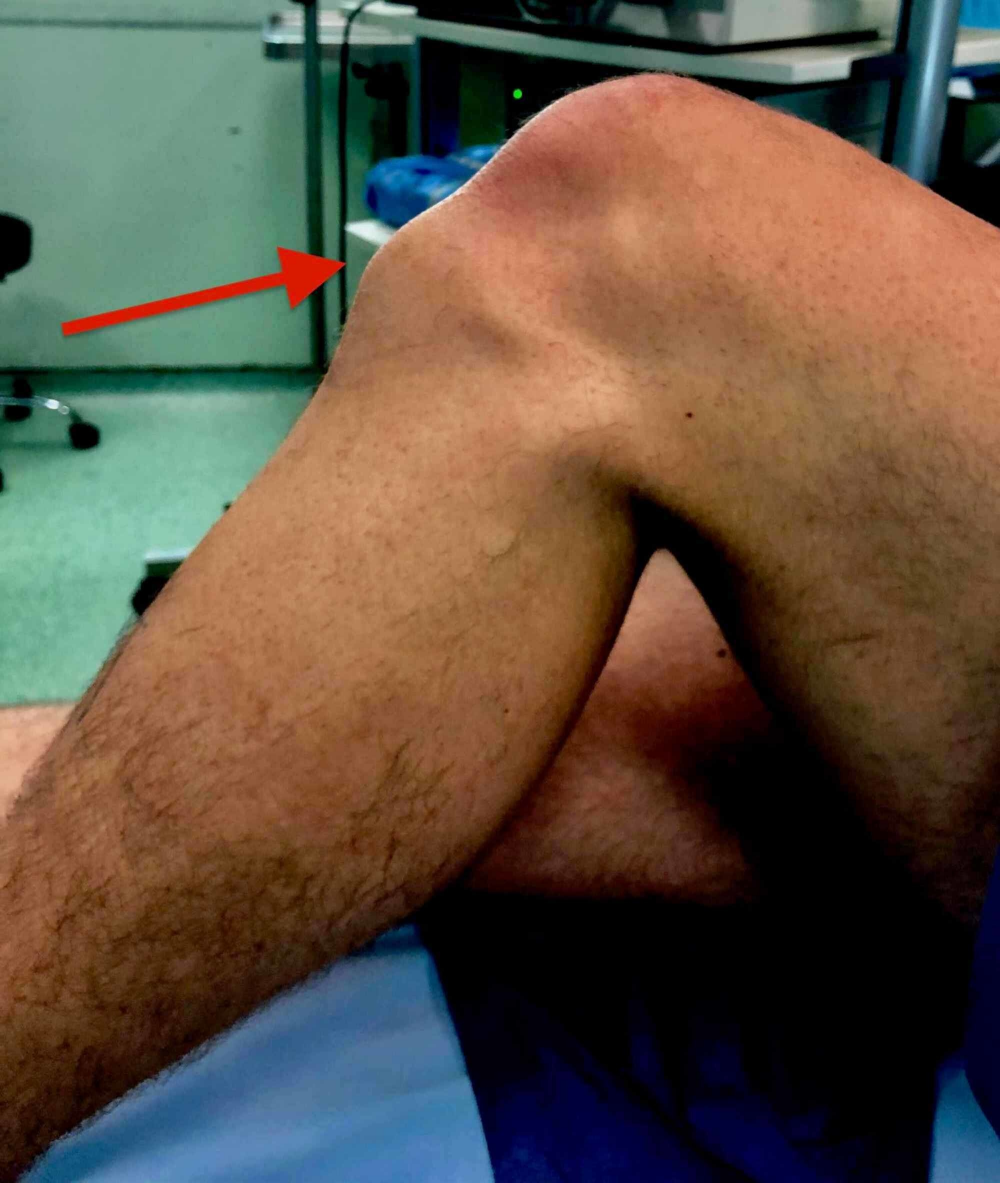
The tibial tuberosity prominence.

**Figure 2:**
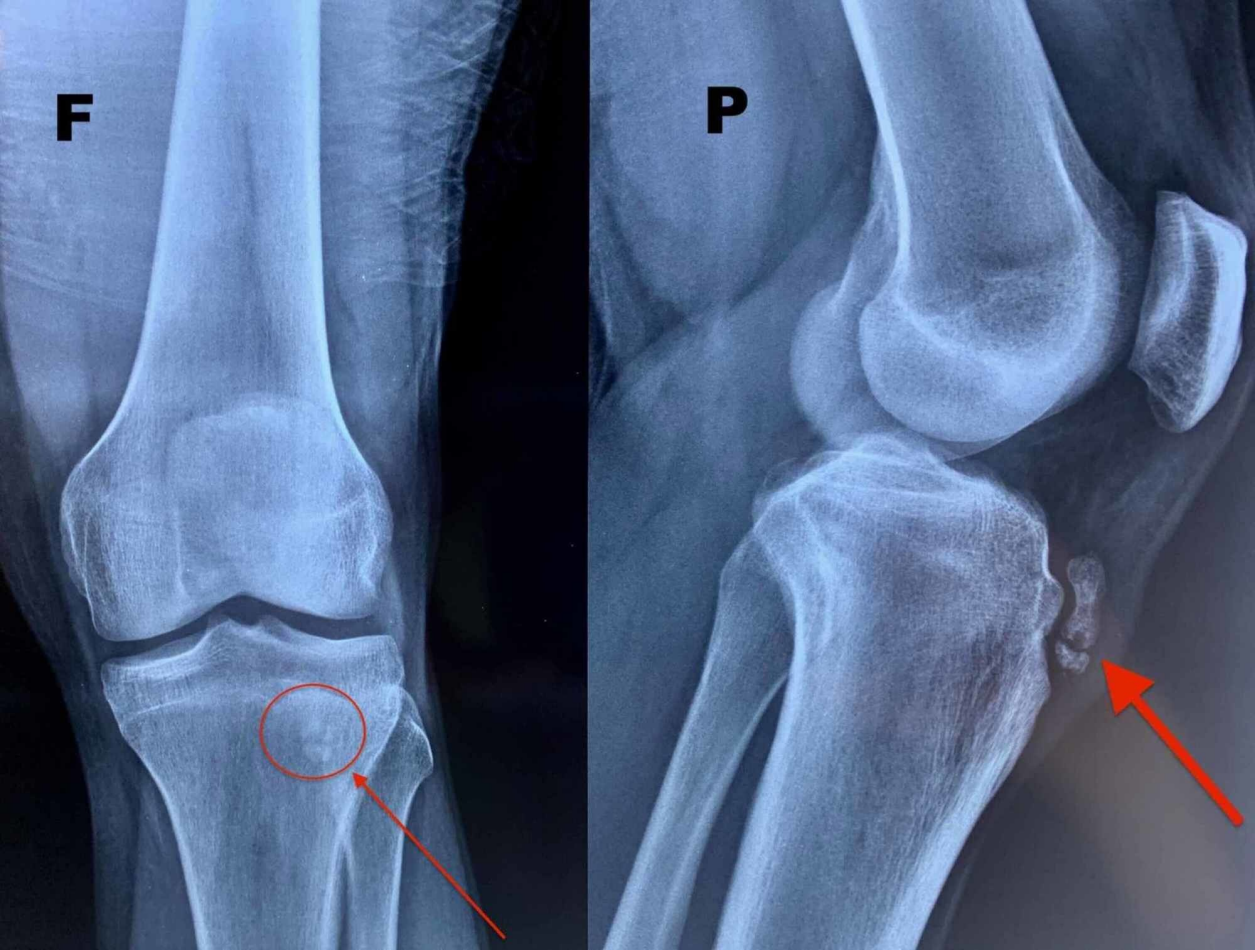
The X-ray in face (F) and profile (P) planes showing the ununited ossicle.

**Figure 3:**
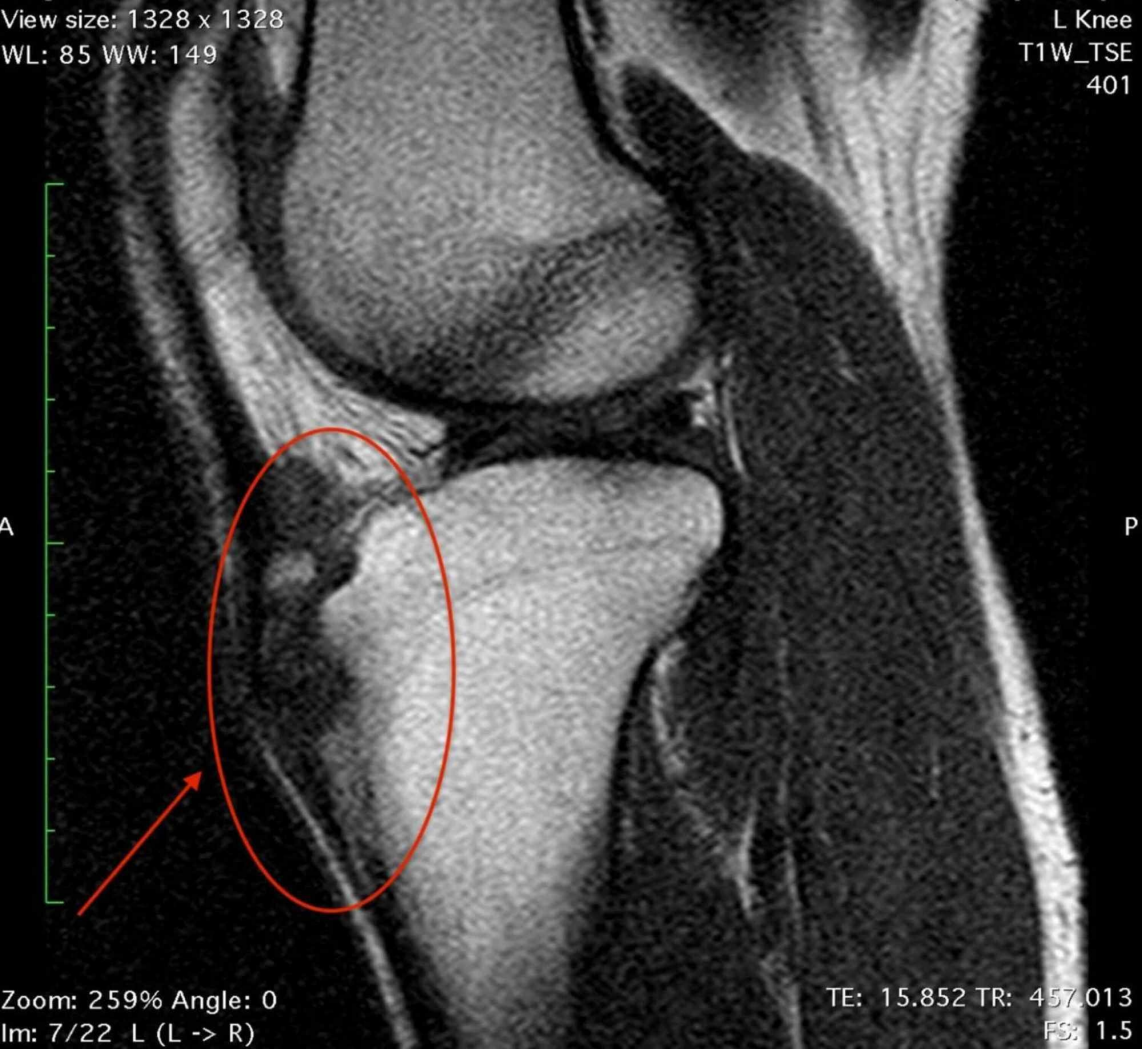
MRI scan showing the ossicle.

A 26-year-old, male semiprofessional soccer player had a prominence of tibial tuberosity for 10 years and reported anterior knee pain during sports activity and while climbing stairs. He claims pain commencement about 15 years ago and since then he mentions occasional pain episodes. He underwent conservative treatment for the last two years with anti-inflammatory drugs and physiotherapy and he had three injections of corticosteroids at different times. On physical examination, there was a permanent tibial tubercle with pain during palpation (Figure 1). Radiographic examination showed an ununited ossicle beneath the patellar tendon (Figure 2). Magnetic resonance imaging sections showed tendinitis on the patellar tendon and an ununited ossicle anterior to the tibial tubercle (Figure 3). Dimensions of the ossicle were 17 mm x 8 mm.

**Figure 4:**
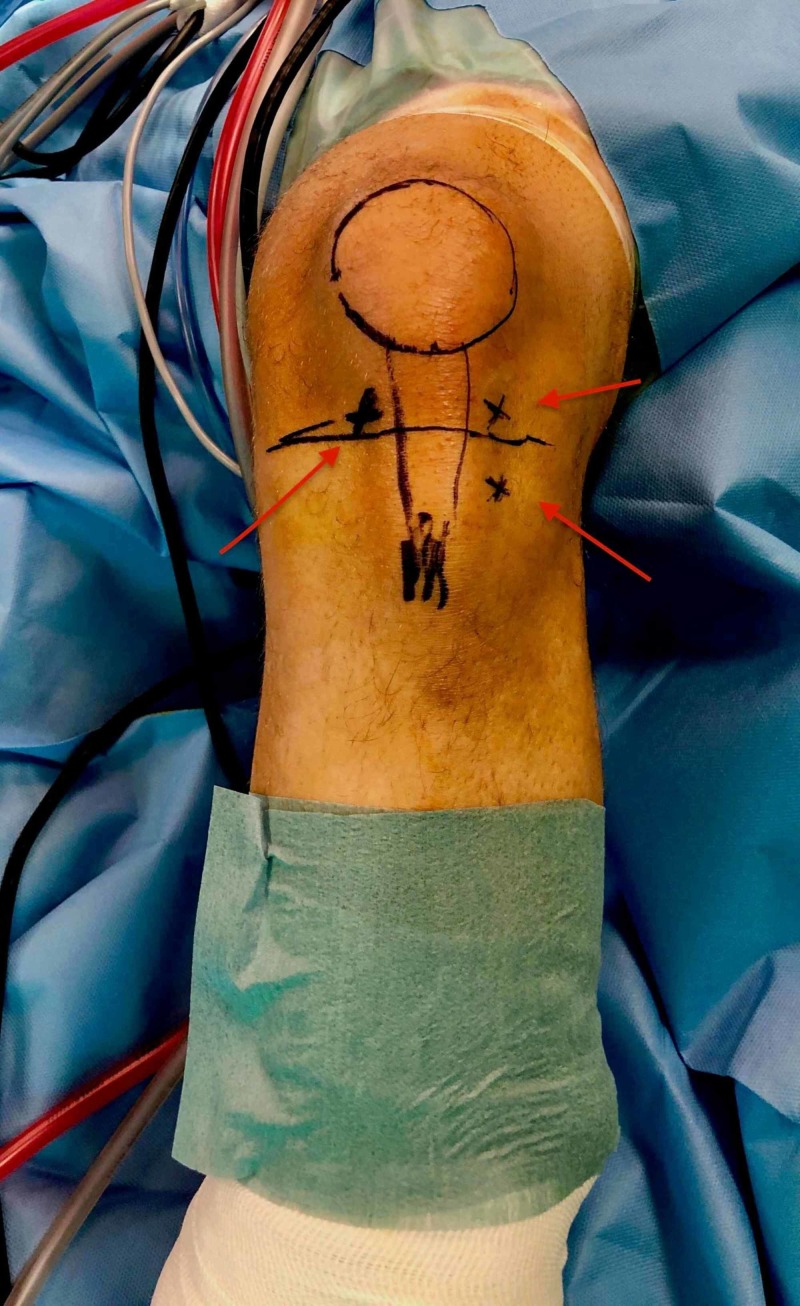
Marking of the arthroscopic portals in referral to the patellar, the patellar tendon and the tibial tuberosity prominence.

**Figure 5:**
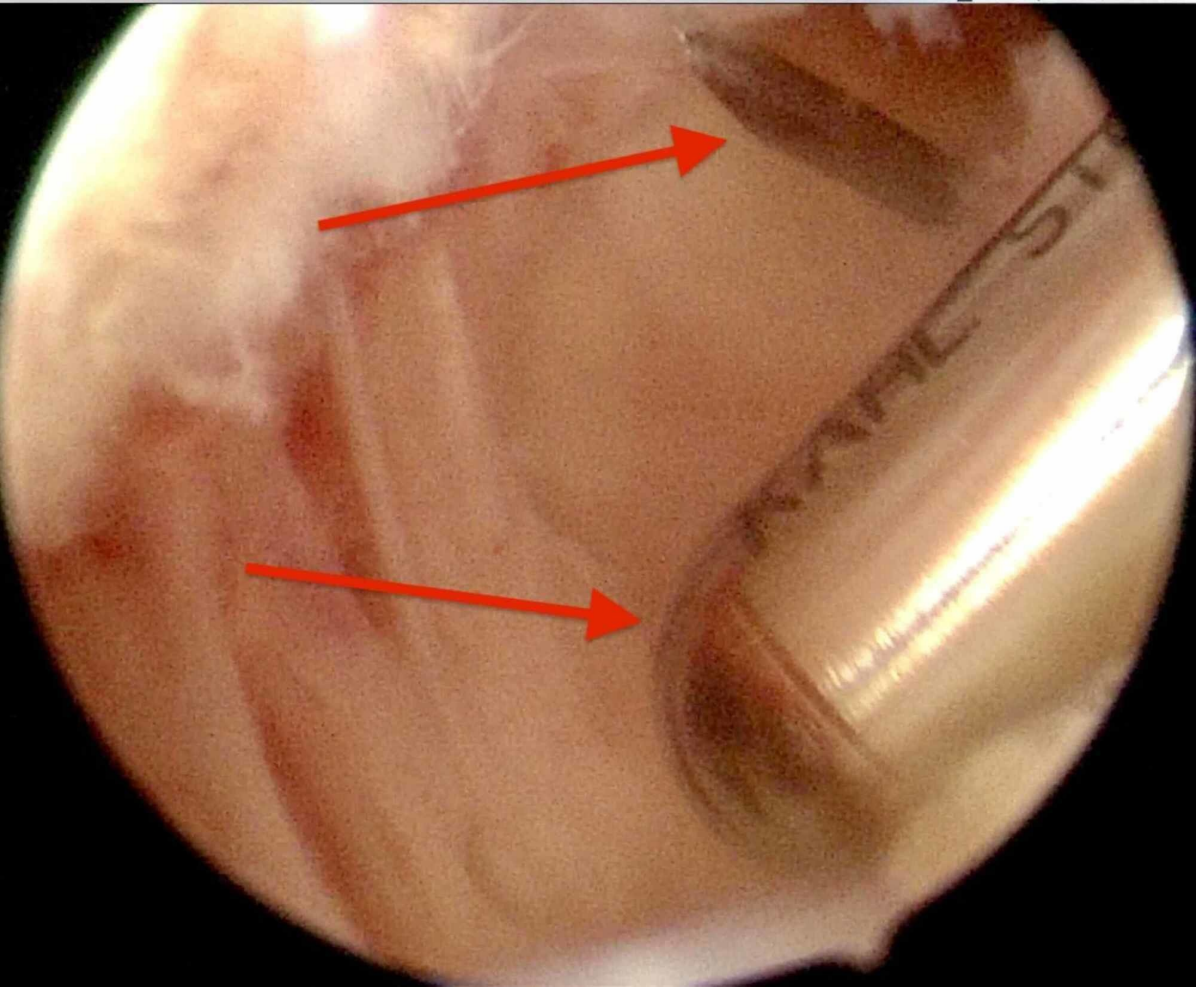
Visualization of the needle used as a guide. Also the saver working on the intra-articular side of the ossicle can be visualized.

**Figure 6:**
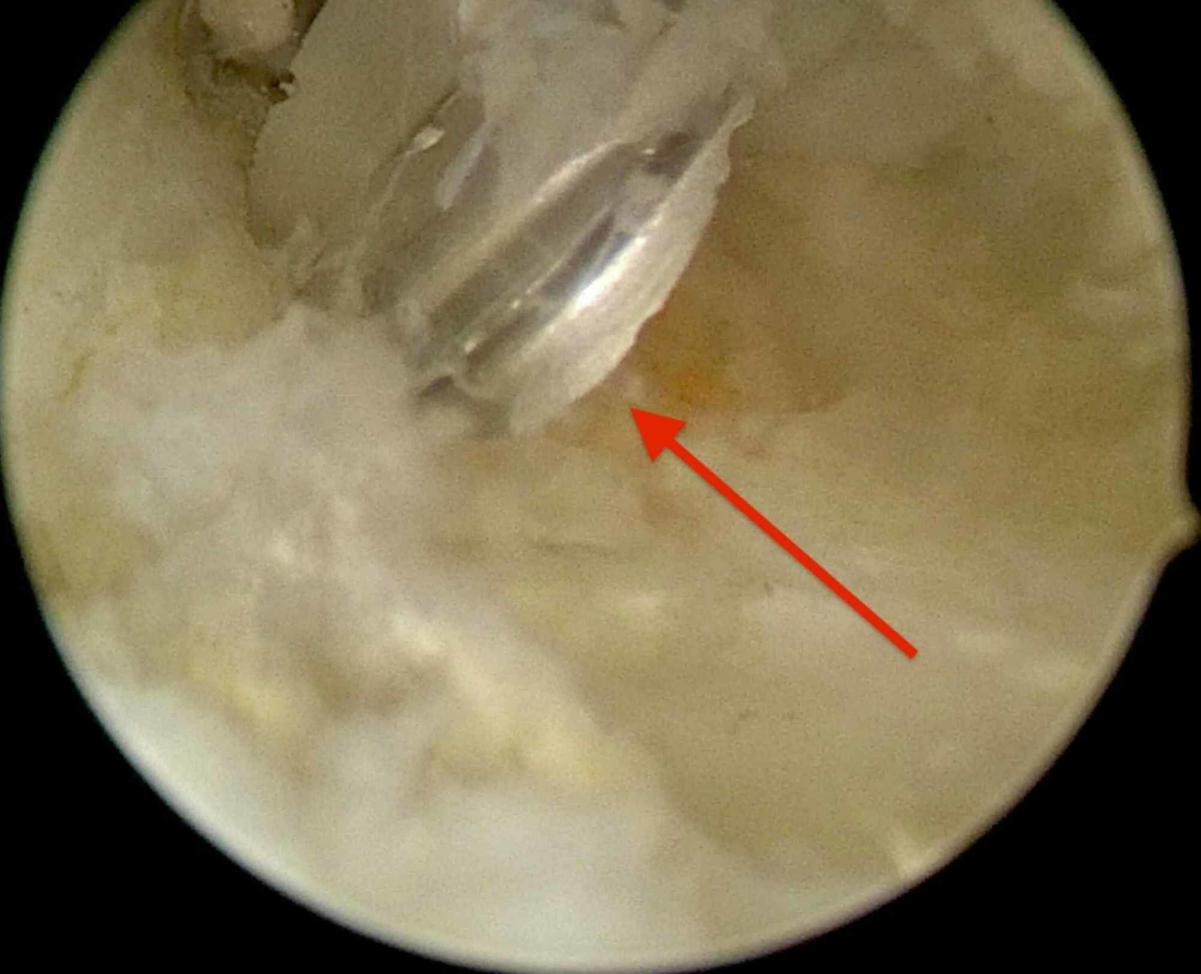
The motorized semi-hooded barrel burr used to resect most of the ossicle.

**Figure 7:**
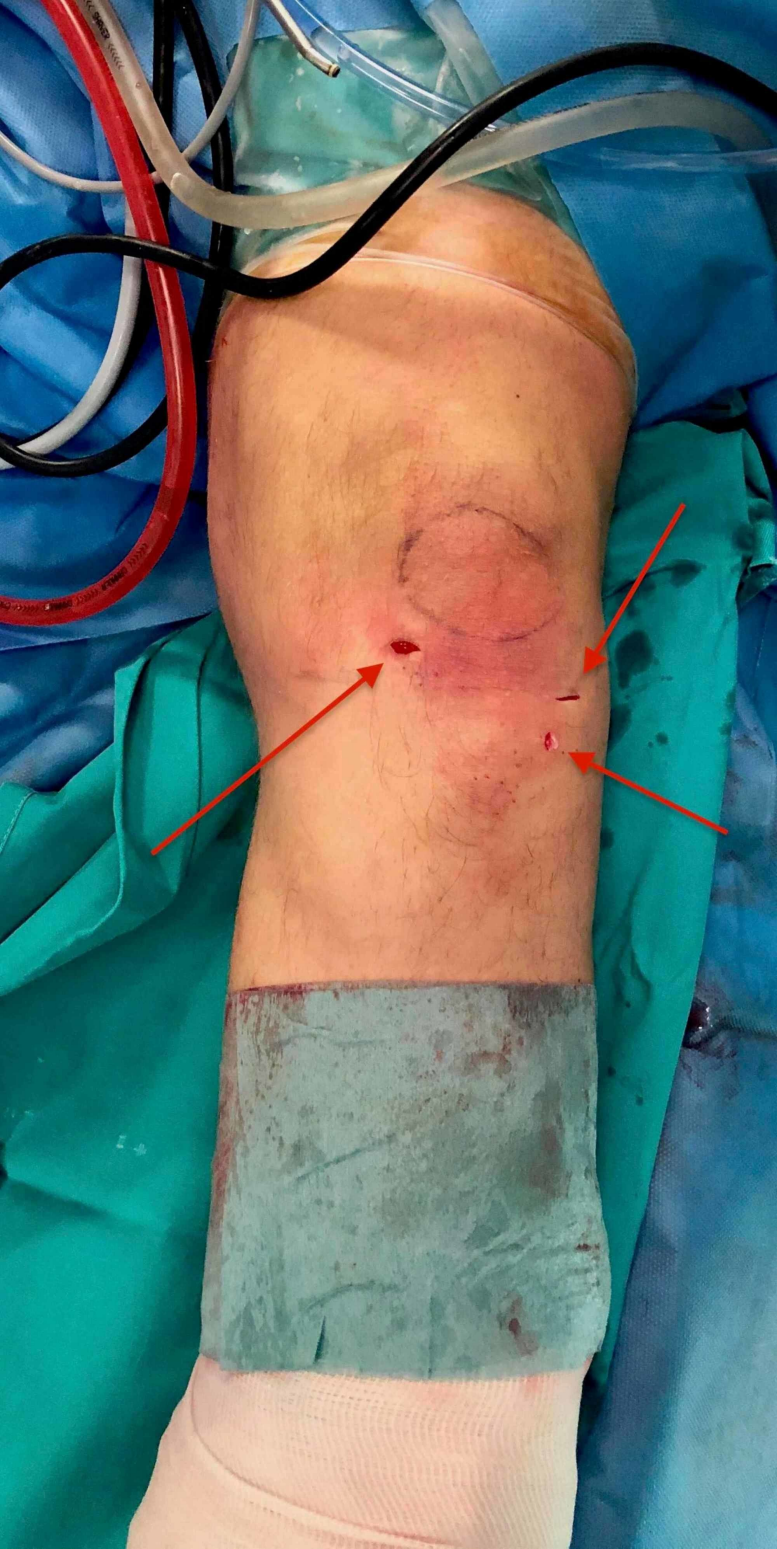
The incisions created and used for arthroscopy and removal of the lesion.

**Figure 8:**
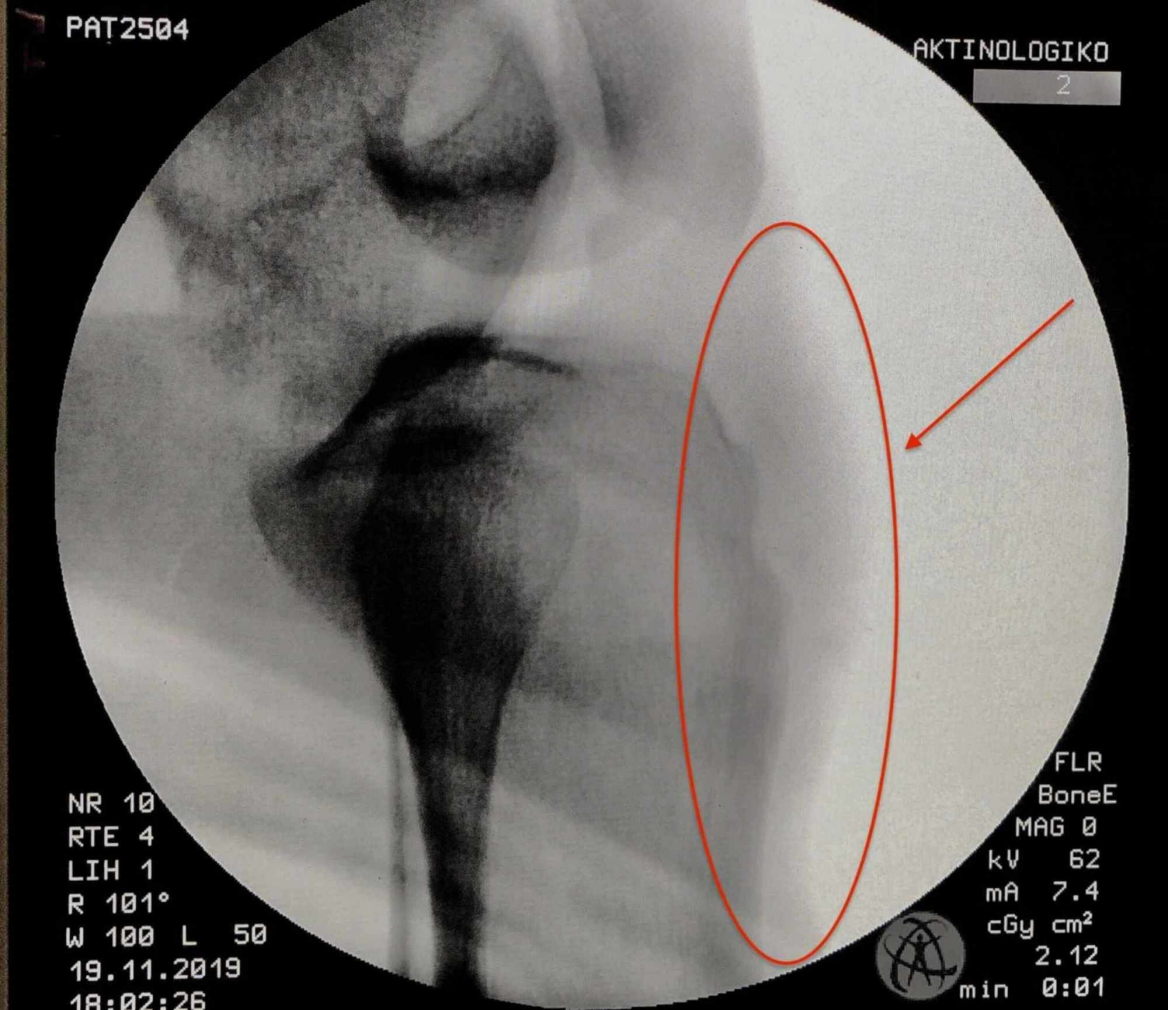
Fluoroscopic control intraoperatively after the ossicle removal.

Diagnostic arthroscopy was performed using a low anterolateral portal close to patellar tendon (Figure 4). The intra-articular arthroscopic examination of the knee was unremarkable and no concomitant abnormality was noticed. An anteromedial portal close to the patellar tendon was used and the infrapatellar fat pad beneath the patellar tendon was debrided with a motorized shaver. Localization of the ununited ossicle was performed by use of an 18-gauge needle that was advanced through the insertion site of the patellar tendon on the tibial tubercle under fluoroscopic control. The needle and the adjacent ossicle were visualized arthroscopically (Figure 5). In order for a better and adequate arthroscopic visualization to be achieved, a multidirectional arthroscope (ENDOCAMELEON® ARTHRO HOPKINS® Telescope) was used at 70°. The knee was extended in order to maximize the working space by relaxing the patellar tendon. The ossicle was identified, embedded in the patellar tendon and the dissection of the ossicle began with debridement of its proximal aspect with a radiofrequency device. Then the removal of the ossicle performed gradually with the use of a motorized shaver (Figure 5) and a motorized semi-hooded barrel burr (Figure 6). In order to achieve an even better approach to the ossicle, a second low anterolateral assisting portal was used. This was created below the first anterolateral portal, just lateral to the patellar tendon and to the ossicle (Figure 7). Fluoroscopic control was performed after removal of the ossicle to ensure no residue of bone (Figure 8).

**Figure 9:**
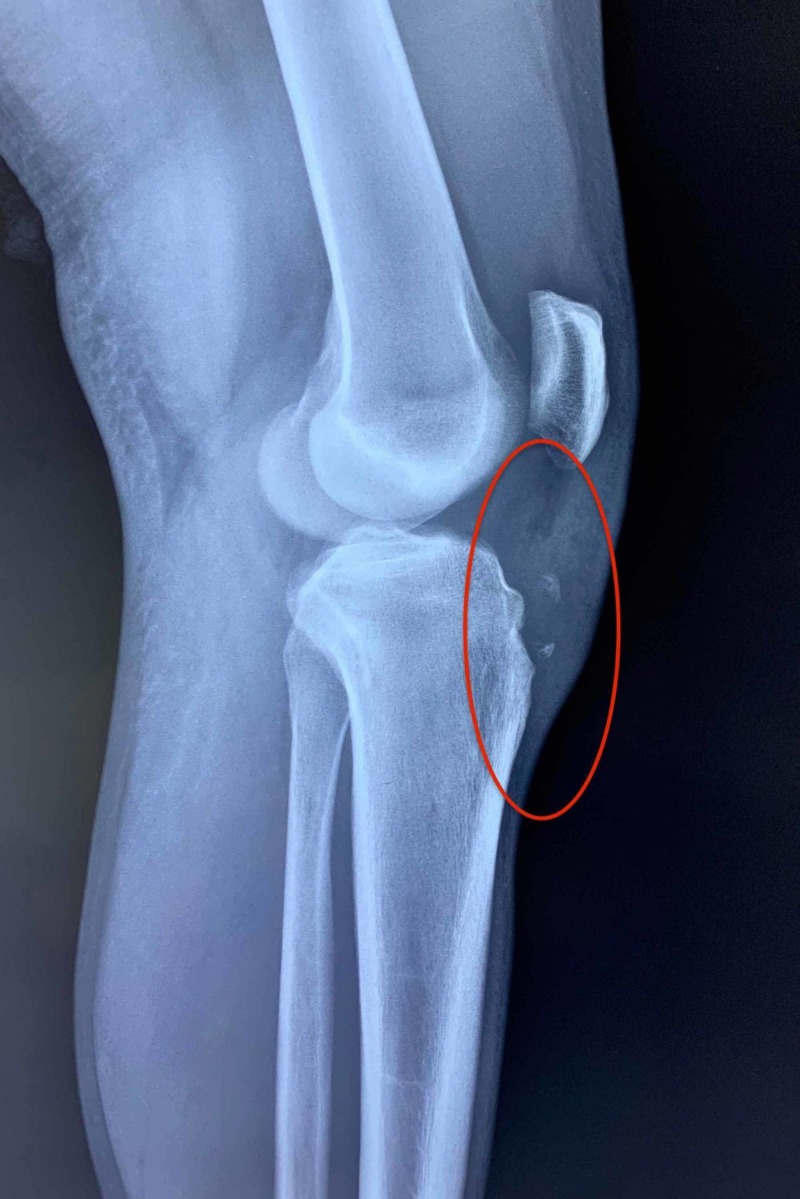
Postoperative X-ray.

**Figure 10:**
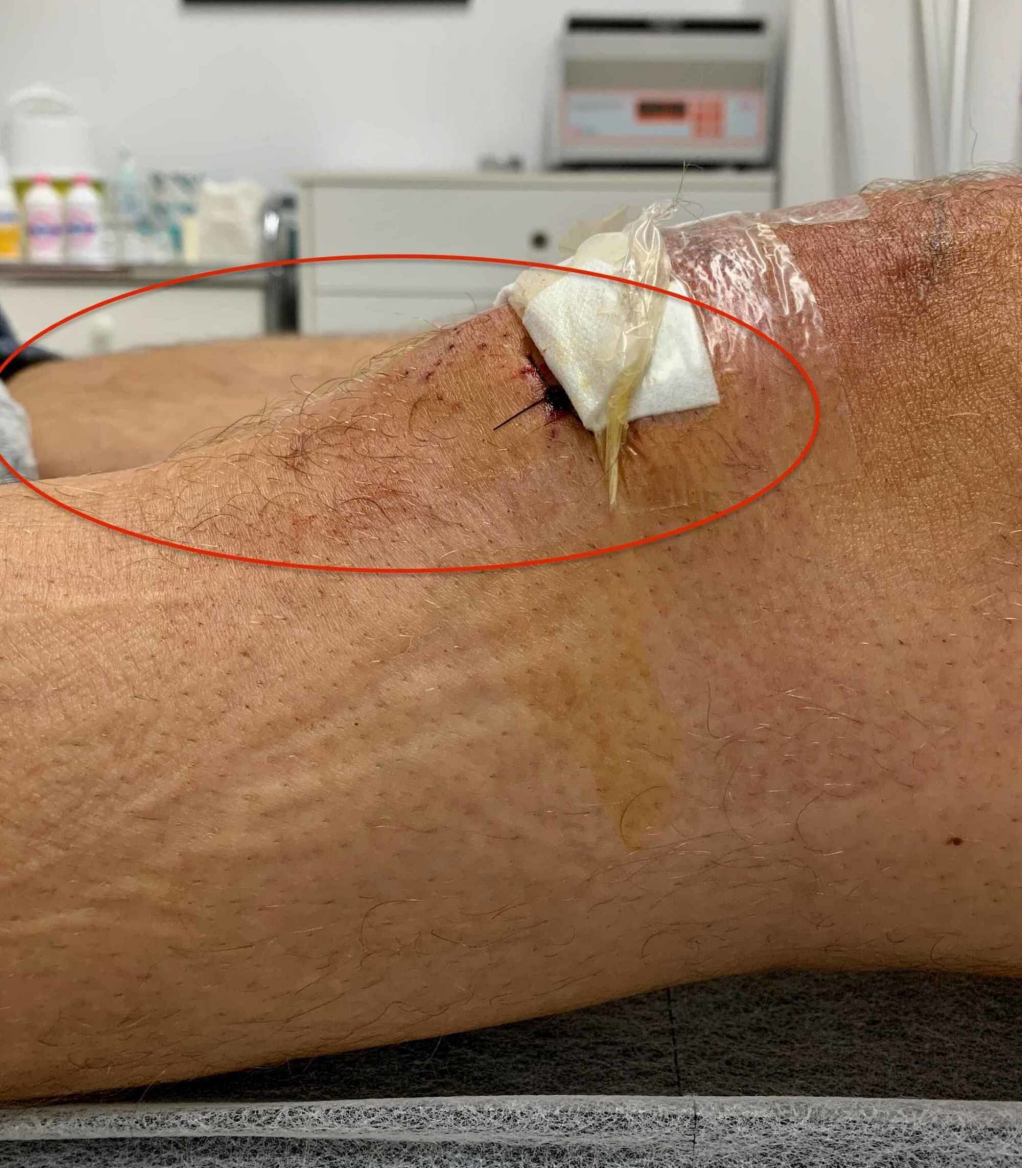
No clinical prominence postoperatively.

Postoperatively, instructions for partial weight bearing and isometric flexion extension 0°-90° exercises for two weeks were given. After two weeks walking without crutches was allowed while no squatting for six weeks was suggested. After two months, the patient returned to sport activities without any restrictions and complaints. Postoperative X-ray showed minimal ossicle residual, and clinically there was no prominence (Figures 9, 10).

## Discussion

Although the natural history of Osgood-Schlatter lesions is that most resolve and it is usually considered to be self-limited, some patients may still have some pain and prominence with kneeling [14]. Krause et al. reported that of 50 patients with an average of nine years follow-up, 60% were still unable to kneel without pain or discomfort and 24% had additional continuing symptoms [5]. Ross and Villard reported that athletes with a history of Osgood-Schlatter disease had higher levels of disability on both daily living and sports activity scales [15]. Conservative measures are regarded to be the treatment of choice in adolescents with Osgood-Schlatter disease. The goals of conservative treatment are to lessen the stress on the tibial tubercle and to reduce the tension in the quadriceps muscle [12].

However, if conservative treatment fails, operative treatment may be considered. There are surgical procedures that include drilling of the tubercle, removal of the loose fragments, autogenous bone peg insertion through the tubercle, tibial tuberosity excision, or sequestrectomy [12]. Furthermore, arthroscopic techniques for surgical treatment of Osgood-Schlatter disease have also been recommended as well as bursoscopic excision [6,12,13]. Arthroscopic procedure is a less invasive surgical modality while the patellar tendon is not violated and the patient avoids an incision directly over the patellar tendon that can cause pain with kneeling [6]. Bursoscopic excision allegedly has advantages over the classical arthroscopic approach because there is no need to perform an anterior interval release and there is no violation of the infrapatellar fat pad. Also a possible meniscus or intermeniscal ligament damage can be avoided [6,13]. However with this approach the working space might be limited and the reduce of the prominence of the tibial tuberosity might be inadequate.

## Conclusions

In the case presented here, the use of multidirectional camera provided excellent view of the ossicle while its localization with a needle proved extremely helpful. The use of the assisting low anterolateral portal and of the motorized semi-hooded barrel burr provided an easy and rapid removal of the larger part of the bony lesion. We believe that the arthroscopic removal of an unresolved Osgood-Schlatter lesion showed excellent results and might be the most appropriate treatment for this condition.
